# A Randomized, Double-Blind, Midazolam-Controlled Trial of Low-Dose Ketamine Infusion in Patients With Treatment-Resistant Depression and Prominent Suicidal Ideation

**DOI:** 10.1093/ijnp/pyad014

**Published:** 2023-03-26

**Authors:** Tung-Ping Su, Cheng-Ta Li, Wei-Chen Lin, Hui-Ju Wu, Shih-Jen Tsai, Ya-Mei Bai, Wei-Chung Mao, Pei-Chi Tu, Li-Fen Chen, Wei-Chi Li, Mu-Hong Chen

**Affiliations:** Department of Psychiatry, Taipei Veterans General Hospital, Taipei, Taiwan; Division of Psychiatry, Faculty of Medicine, National Yang Ming Chiao Tung University, Taipei, Taiwan; Department of Medical Research, Taipei Veterans General Hospital, Taipei, Taiwan; Institute of Brain Science, National Yang Ming Chiao Tung University, Taipei, Taiwan; Department of Psychiatry, Cheng Hsin General Hospital, Taipei, Taiwan; Division of Psychiatry, Faculty of Medicine, National Yang Ming Chiao Tung University, Taipei, Taiwan; Institute of Brain Science, National Yang Ming Chiao Tung University, Taipei, Taiwan; Department of Psychiatry, Taipei Veterans General Hospital, Taipei, Taiwan; Division of Psychiatry, Faculty of Medicine, National Yang Ming Chiao Tung University, Taipei, Taiwan; Institute of Brain Science, National Yang Ming Chiao Tung University, Taipei, Taiwan; Department of Psychiatry, Taipei Veterans General Hospital, Taipei, Taiwan; Department of Psychiatry, Taipei Veterans General Hospital, Taipei, Taiwan; Division of Psychiatry, Faculty of Medicine, National Yang Ming Chiao Tung University, Taipei, Taiwan; Institute of Brain Science, National Yang Ming Chiao Tung University, Taipei, Taiwan; Department of Psychiatry, Taipei Veterans General Hospital, Taipei, Taiwan; Division of Psychiatry, Faculty of Medicine, National Yang Ming Chiao Tung University, Taipei, Taiwan; Institute of Brain Science, National Yang Ming Chiao Tung University, Taipei, Taiwan; Department of Psychiatry, Taipei Veterans General Hospital, Taipei, Taiwan; Department of Psychiatry, Cheng Hsin General Hospital, Taipei, Taiwan; Division of Psychiatry, Faculty of Medicine, National Yang Ming Chiao Tung University, Taipei, Taiwan; Department of Medical Research, Taipei Veterans General Hospital, Taipei, Taiwan; Department of Psychiatry, Taipei Veterans General Hospital, Taipei, Taiwan; Division of Psychiatry, Faculty of Medicine, National Yang Ming Chiao Tung University, Taipei, Taiwan; Institute of Brain Science, National Yang Ming Chiao Tung University, Taipei, Taiwan; Department of Psychiatry, Taipei Veterans General Hospital, Taipei, Taiwan; Institute of Brain Science, National Yang Ming Chiao Tung University, Taipei, Taiwan; Institute of Biomedical Informatics, National Yang Ming Chiao Tung University, Taipei, Taiwan; Integrated Brain Research Unit, Department of Medical Research, Taipei Veterans General Hospital, Taipei, Taiwan; Institute of Brain Science, National Yang Ming Chiao Tung University, Taipei, Taiwan; Institute of Biomedical Informatics, National Yang Ming Chiao Tung University, Taipei, Taiwan; Integrated Brain Research Unit, Department of Medical Research, Taipei Veterans General Hospital, Taipei, Taiwan

**Keywords:** Treatment-resistant depression, prominent suicidal ideation, ketamine, Taiwan

## Abstract

**Background:**

The benefits of low-dose ketamine for patients with treatment-resistant depression (TRD) and prominent suicidal ideation require further investigation. The effects of treatment refractoriness, the duration of the current depressive episode, and the number of prior antidepressant failures on ketamine efficacy also require clarification.

**Methods:**

We recruited 84 outpatients with TRD and prominent suicidal ideation—defined as a score ≥4 on item 10 of the Montgomery–Åsberg Depression Rating Scale (MADRS)—and randomized them into 2 groups to receive 0.5 mg/kg ketamine or 0.045 mg/kg midazolam. We assessed depressive and suicidal symptoms prior to infusion; 240 minutes post infusion; and 2, 3, 5, 7, and 14 days post infusion.

**Results:**

According to the MADRS scores, the antidepressant effect (*P* = .035) was significantly noted in the ketamine group up to 14 days than in the midazolam group. However, the antisuicidal effect of ketamine, as measured by the Columbia-Suicide Severity Rating Scale Ideation Severity Subscale (*P* = .040) and MADRS item 10 (*P* = .023), persisted only 5 days post infusion. Furthermore, the antidepressant and antisuicidal effects of ketamine infusion were noted particularly in patients whose current depressive episode lasted <24 months or whose number of failed antidepressants was ≤4.

**Conclusions:**

Low-dose ketamine infusion is a safe, tolerable, and effective treatment for patients with TRD and prominent suicidal ideation. Our study highlights the importance of timing; specifically, ketamine is more likely to achieve therapeutic response when the current depressive episode lasted <24 months and the number of failed antidepressants is ≤4.

**Significance Statement**

Low-dose ketamine infusion had rapid antidepressant and antisuicidal effects among patients with TRD and prominent suicidal ideation. In addition, the antidepressant effect of ketamine persisted for up to 2 weeks, but the antisuicidal effect lasted only 5 days. Furthermore, timing of ketamine treatment is crucial; specifically, patients with current depressive episodes that have persisted <24 months or ≤4 failed antidepressant treatments may receive the greatest benefits from low-dose ketamine infusion.

## INTRODUCTION

Over the last half century, worldwide suicide rates have increased by 60%, and suicide accounted for more than 1 million deaths in 2020 (Bertolote and Fleischmann, 2002; Fazel et al., 2013). The World Health Organization named Taiwan as one of the countries with the highest prevalence (>13/100 000 person-years) of suicide worldwide (Bertolote and Fleischmann, 2002; Fazel et al., 2013). The prevalence of suicide in Taiwan peaked in 2006 (19.3/100 000 person-years) and remained at approximately 16/100 000 person-years from 2012 to 2019 despite the implementation of the Taiwan Suicide Prevention Program in 2005 (Chen, Yang et al., 2021).

A growing body of evidence has supported the rapid and sustained antisuicidal effect of low-dose ketamine (Abbar et al., 2022). A randomized, double-blind, placebo-controlled trial of patients with severe suicidal ideation (Scale for Suicidal Ideation score >3) revealed a greater proportion of patients achieving complete remission in the ketamine group (46 of 83; 63.0%) than in the control group (25 of 73; 31.6%) on day 3 (Abbar et al., 2022). In this study by Abbar et al., only 40% of participants with severe suicidal ideation were experiencing a major depressive episode, and less than 10% had comorbid dysthymia. The low treatment refractoriness of the study patients may partially explain why suicidal ideation remained in remission at week 6 after ketamine infusion in up to 60% of the participants (Abbar et al., 2022). Feeney et al. reported that the initial antisuicidal effect of a single ketamine infusion rapidly diminished in patients with treatment-resistant depression (TRD), losing efficacy as early as day 3 (Feeney et al., 2021). However, they reported low baseline scores of suicidal ideation—2.90 ± 0.74 on the Montgomery–Åsberg Depression Rating Scale (MADRS) suicide item (item 10)—which may have affected their results (Feeney et al., 2021). A meta-analysis of 10 randomized placebo-controlled trials involving 167 patients with major depression, bipolar depression, or posttraumatic stress disorder reported that ketamine rapidly (within 1 day) and significantly reduced suicidal ideation, according to both clinician-administered and self-report outcome measures (Wilkinson et al., 2018); the effect sizes were moderate to large (Cohen *d *= .48–.85) at all time points following administration (days 1–7) (Wilkinson et al., 2018).

In a real-world clinical setting, however, TRD and severe suicidal ideation commonly occur together and exacerbate the effects of one another (Montgomery, 1980; Al-Harbi, 2012). Ionescu et al. observed a lack of antidepressant and antisuicidal effects after a ketamine infusion of 0.5 mg/kg among patients with TRD and chronic suicidal ideation (Ionescu et al., 2019). Our previous clinical trial revealed that severe treatment refractoriness may affect the therapeutic efficacy of ketamine infusion (Chen Lin et al., 2021a). Accordingly, optimizing the timing of ketamine infusion is vital, and whether the antidepressant and antisuicidal effects of low-dose ketamine can be generalized to patients with severe TRD and prominent suicidal ideation remains unclear.

In the current study, we enrolled 84 patients with TRD and prominent suicidal ideation (MADRS item 10 ≥4) who were randomized to 2 groups receiving a single infusion of either 0.5 mg/kg ketamine or 0.045 mg/kg midazolam. We followed the patients for 2 weeks to assess their depressive and suicidal symptoms. We hypothesized that the low-dose ketamine infusion would exert rapid and sustained antidepressant and antisuicidal effects in patients with TRD and prominent suicidal ideation, particularly among those without severe treatment refractoriness or chronic TRD.

## METHODS

### Inclusion Criteria and Study Procedure

Adult outpatients aged between 20 and 64 years who were diagnosed with major depressive disorder based on the Diagnostic and Statistical Manual of Mental Disorders, Fifth Edition, with inadequate response to at least 2 different antidepressants with adequate dosage and treatment duration and had a prominent suicidal ideation were enrolled in current study. Participants were randomized into 2 groups receiving a single infusion of either 0.5 mg/kg ketamine or 0.045 mg/kg midazolam (Grunebaum et al., 2018). The prominent suicidal ideation was defined by scores ≥4 on the MADRS item 10 (Montgomery, 1980). Depressive symptoms were examined using the MADRS immediately prior to infusion; at 40, 80, and 240 minutes post infusion; and sequentially, on days 2, 3, 5, 7, and 14 post infusion (Montgomery, 1980). Treatment response was defined based on at least 1 MADRS score of a ≥50% reduction at day 2 or day 3 post infusion. Suicide symptoms were assessed using the 5 yes or no questions, including question 1: wish to be dead; question 2: nonspecific suicidal thoughts; and questions 3–5: more specific suicidal thoughts and intent to act, on Columbia-Suicide Severity Rating Scale—Ideation Severity Subscale (CSSRS-ISS) prior to infusion; at 240 minutes post infusion, and sequentially on days 2, 3, 5, 7, and 14 post infusion (Posner et al., 2011). Suicide symptoms were additionally examined by the self-reported Positive and Negative Suicide Ideation Inventory (PANSI) at baseline; at 240 minutes post infusion; and sequentially on days 2, 3, 7, and 14 post infusion (Osman et al., 1998). Level of treatment refractoriness was defined by the Maudsley staging method and was divided into low (≤7), moderate (8–10), and high (≥11) scores (Fekadu et al., 2009). Exclusion criteria included major medical or neurological diseases or a history of alcohol or substance use disorders in current study. This study accorded with the Declaration of Helsinki and was approved by the institutional review boards of Taipei Veterans General Hospital and Cheng Hsin General Hospital. All participants gave their written informed consent. Clinical trial registration: UMIN Clinical Trials Registry (UMIN-CTR): registration number: UMIN000033916 and UMIN000033760.

### Statistical Methods

Continuous variables and nominal variables were analyzed using 1-way ANOVA and Fisher’s chi square tests, respectively, to assess differences between the 2 infusion groups (0.5 mg/kg ketamine or 0.045 mg/kg midazolam) with respect to demographic and clinical data. The generalized estimating equation (GEE) models with the autoregressive method for correlations of repeated measures for the same individual over time and with the adjustment of age, sex, and baseline clinical symptoms were used to assess the trajectories of total MADRS, MADRS item 10, total CSSRS-ISS, and PANSI-Positive Suicide Ideation (PSI) and Negative Suicide Ideation (NSI) scores during the study period with the infusion group (0.5 mg/kg ketamine vs 0.045 mg/kg midazolam) as a between-patient factor and time (baseline, infusion, and follow-up) as a within-patient factor as well as all possible interactions. In addition, we examined the roles of 3 clinical factors, including treatment refractoriness (low and moderate vs high), duration of current episode (<24 vs ≥24 months), and failure numbers of antidepressants (≤4 vs >4), on the trajectories of the above clinical symptoms. Furthermore, owing to the evidence that the duration of the antisuicidal effect of ketamine infusion may not be longer than 1 week (Murrough et al., 2015), additional analyses with a follow-up duration <7 days were performed. Two-tailed *P* < .05 was considered statistically significant. All data processing and statistical analyses were performed using SPSS, version 17 (SPSS Inc., Chicago, IL).

## RESULTS

The study flowchart is shown in supplementary Figure 1. In all, 90 outpatients with TRD and prominent suicidal ideation were screened; 6 patients were excluded owing to hypertension (n = 3), type 2 diabetes mellitus (n = 1), and refusal of written informed consent (n = 2). Finally, 84 patients were randomly assigned to a single infusion of either 0.5 mg/kg ketamine or 0.045 mg/kg midazolam.

Table 1 showed no difference in age (*P* = .351), sex (*P* = .634), body mass index (*P* = .186), age at illness onset (*P* = .864), history of attempted suicide (*P *> .738), and treatment refractoriness (*P* = .788) between the 2 infusion groups. The midazolam group had slightly higher total MADRS scores (38.26 ± 3.83 vs 35.83 ± 4.53, *P* = .010) at baseline than the ketamine group (Table 1). The baseline scores of MADRS item 10 (*P* = .440), CSSRS-ISS (*P* = .684), PANSI-PSI (*P* = .746), and PANSI-NSI (*P* = .549) did not differ between groups (Table 1). In addition, the psychiatric comorbidities, including posttraumatic stress disorder (*P* > .999), panic disorder (*P* > .999), and generalized anxiety disorder (*P* = .405), did not differ between groups (Table 1).

**Table 1.** Demographic Characteristics, Baseline Clinical Symptoms, and Treatment Outcomes of Patients With TRD and Prominent Suicidal Ideation Receiving a Single Infusion of Ketamine vs Midazolam Placebo

	Patients with TRD having prominent suicidal ideation		
	Ketamine group (n = 42)	Midazolam group (n = 42)	*P* alue
Age (SD), y	34.26 (13.34)	36.88 (12.21)	.351
Female, n (%)	28 (66.7)	31 (73.8)	.634
BMI (SD)	25.48 (6.21)	23.85 (4.95)	.186
Education (SD), y	14.43 (2.39)	14.74 (2.72)	.581
Age at illness onset (SD), y	24.07 (9.71)	24.43 (9.31)	.864
History of attempted suicide, n (%)	36 (85.7)	38 (90.5)	.738
No. of prior suicidal attempt, n (%)			.109
Never	6 (14.3)	4 (9.6)	
1	21 (50.0)	14 (33.3)	
2-4	5 (11.9)	14 (33.3)	
≥5	10 (23.8)	10 (23.8)	
MSM, total scores (SD)	9.86 (1.97)	10.38 (1.64)	.189
Treatment refractoriness, n (%)			.788
Low	4 (9.5)	4 (9.5)	
Moderate	19 (45.2)	16 (38.1)	
High	19 (45.2)	22(52.4)	
Current episode, n (%)			.495
<24 mo	17 (40.5)	13 (31.0)	
≥24 mo	25 (59.5)	29 (69.0)	
Failure no. of antidepressants, n (%)			.379
≤4	21 (50.0)	26 (61.9)	
>4	21 (50.0)	16 (38.1)	
Clinical symptoms at baseline (SD)			
MADRS	35.83 (4.53)	38.26 (3.83)	.010
MADRS item 10	4.19 (0.40)	4.26 (0.45)	.440
CSSRS-ISS	3.02 (0.81)	3.10 (0.79)	.684
PANSI-PSI	12.21 (4.87)	11.88 (4.53)	.746
PANSI-NSI	30.45 (7.06)	29.55 (6.71)	.549
Psychiatric comorbidities, n (%)			
PTSD	11 (26.2)	11 (26.2)	>.999
Panic disorder	27 (64.3)	26 (61.9)	>.999
Generalized anxiety disorder	32 (76.2)	36 (85.7)	.405
Treatment response based on at least once MADRS scores of ≥50% reduction at d 2 or 3 post infusion			**.020**
Response, n (%)	15 (35.7)	5 (11.9)	
Nonresponse, n (%)	27 (64.3)	37 (88.1)	
Remission of suicidal ideation based on total CSSRS-ISS scores = 0 at post infusion			
Day 1, 240 min	14 (33.3)	3 (7.1)	**.005**
Day 2	14 (33.3)	4 (9.5)	**.015**
Day 3	14 (33.3)	3 (7.1)	**.005**
Day 5	11 (26.2)	3 (7.1)	**.038**
Day 7	8 (19.0)	5 (11.9)	.548
Day 14	7 (16.7)	7 (16.7)	>.999
Adverse effects during infusion, n (%)			
Derealization	29 (69.0)	7 (16.7)	<.001
Dizziness	24 (57.1)	5 (11.9)	<.001
Nausea	2 (4.8)	4 (9.5)	.676
Crying	6 (14.3)	0 (0.0)	.026
Somnolence	1 (2.4)	0 (0.0)	>.999

Abbreviations: BMI, body mass index; CSSRS-ISS, Columbia-Suicide Severity Rating Scale-Ideation Severity Subscale; MADRS, Montgomery-Åsberg Depression Rating Scale; MSM, Maudsley Staging Method; NSI, Negative Suicide Ideation; PANSI, Positive and Negative Suicide Ideation Inventory; PSI, Positive Suicide Ideation; PTSD, posttraumatic stress disorder; TRD, treatment-resistant depression.

Bold type indicates the statistical significance.

**Figure 1.**
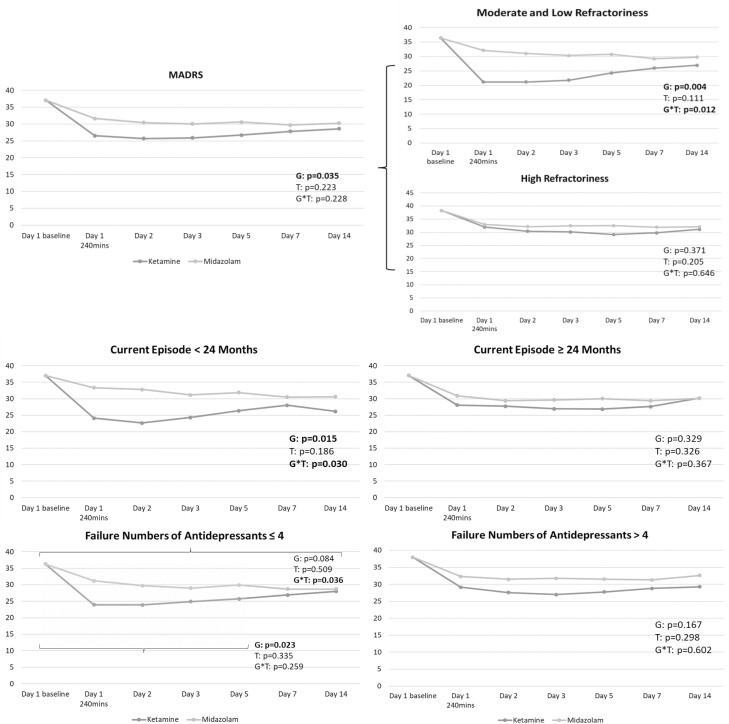
Trajectory of Montgomery-Åsberg Depression Rating Scale (MADRS) scores among patients with treatment-resistant depression (TRD) having prominent suicidal ideation. G, group (ketamine vs midazolam); T, time.

Table 1 indicated the greater treatment response (35.7% vs 11.9%, *P* = .020) in the ketamine group than in the midazolam group. The GEE model reported the significant antidepressant effect (*P* = .035) based on the total MADRS scores up to day 14 in the ketamine group compared with the midazolam group (Figure 1). Stratified by the level of treatment refractoriness, the antidepressant effect of low-dose ketamine was noted in patients with moderate and low refractoriness (*P* = .004) but not in those with high refractoriness (*P* = .371) (Figure 1). In addition, the antidepressant effect of ketamine infusion was noted particularly in patients whose current depressive episode had lasted <24 months (*P* = .015) or in those with ≤4 failed antidepressant treatments (*P* = .023) (Figure 1). Patients with a current episode >24 months (*P* = .329) and those with >4 failed antidepressant treatments (*P* = .167) did not respond to ketamine infusion (Figure 1).

**Figure 2.**
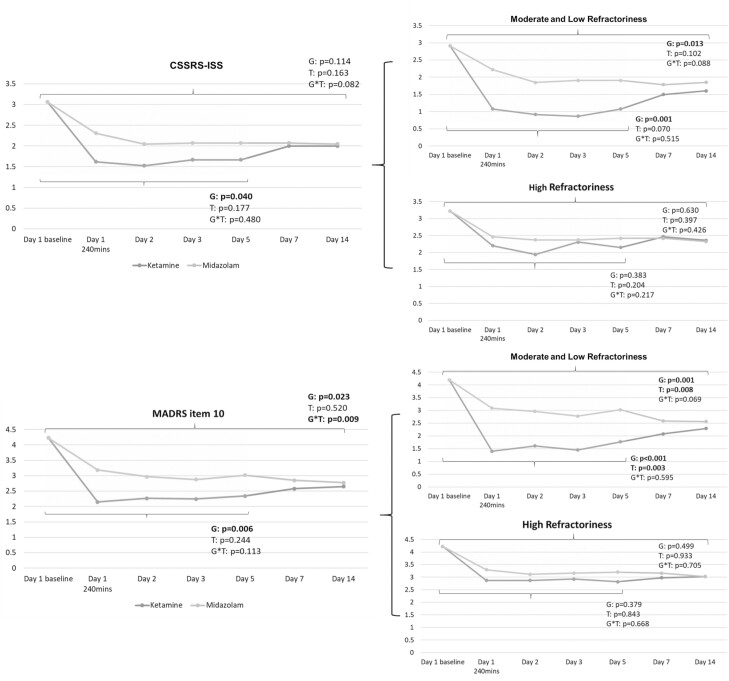
Trajectory of Columbia-Suicide Severity Rating Scale-Ideation Severity Subscale (CSSRS-ISS) and Montgomery-Åsberg Depression Rating Scale (MADRS) item 10 scores among patients with treatment-resistant depression (TRD) having prominent suicidal ideation. G, group (ketamine vs midazolam); T, time. Note: The 2 sets of *P* values refer to day 5 and day 14, respectively.

**Figure 3.**
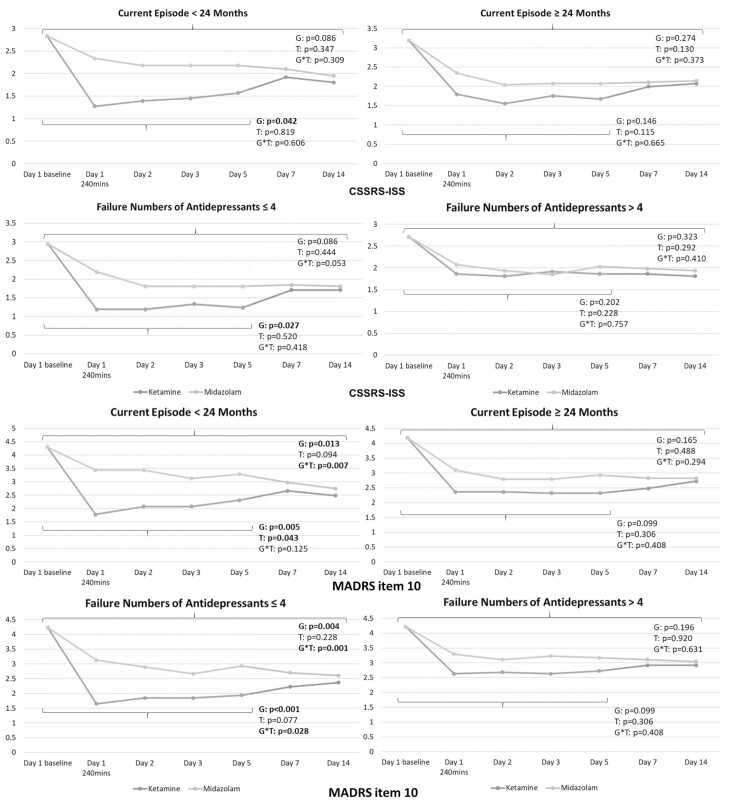
Trajectory of Columbia-Suicide Severity Rating Scale-Ideation Severity Subscale (CSSRS-ISS) and Montgomery-Åsberg Depression Rating Scale (MADRS) item 10 scores among patients with treatment-resistant depression (TRD) having prominent suicidal ideation, stratified by duration of current episode and failure numbers of antidepressants. G, group (ketamine vs midazolam); T, time. Note: The 2 sets of *P* values refer to day 5 and day 14, respectively.

More numbers of patients receiving ketamine reached full remission of suicidal ideation based on the total CSSRS-ISS score = 0 from day 2 (n = 14 vs 4, *P* = .015) to day 5 (n = 11 vs 3, *P* = .038) compared with those receiving midazolam (Table 1). GEE models identified the significant antisuicidal effects of low-dose ketamine infusion measured by the CSSRS-ISS (*P* = .040) and MADRS item 10 (*P* = .023) at least up to day 5 (Figure 2). The antisuicidal effect was noted in patients with moderate and low refractoriness (CSSRS-ISS: *P* = .001; MADRS item 10: *P* < .001) but not in those with high refractoriness (*P* = .383; *P* = .379) (Figure 2). Furthermore, only patients whose current depressive episode had lasted <24 months and those with ≤4 failed antidepressant treatments benefited from the antisuicidal effect of ketamine infusion measured by the CSSRS-ISS (*P* = .042; *P* = .027) and MADRS item 10 (*P* = .005; *P* < .001) (Figure 3).

**Figure 4.**
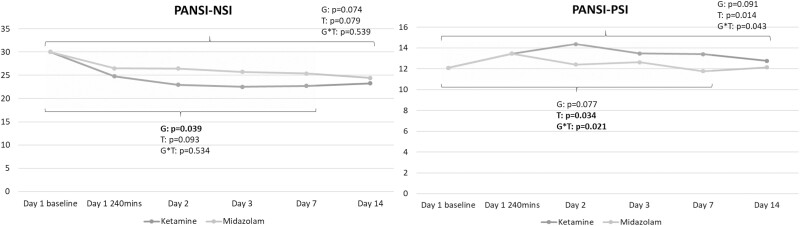
Trajectory of Positive and Negative Suicide Ideation Inventory (PANSI) scores among patients with treatment-resistant depression (TRD) having prominent suicidal ideation. G, group (ketamine vs midazolam); NSI, Negative Suicide Ideation; PSI, Positive Suicide Ideation; T, time. Note: The 2 sets of *P* values refer to day 7 and day 14, respectively.

Figure 4 demonstrated that the significant antisuicidal effect measured by the PANSI-NSI scores persisted for up to day 7 (*P* = .039) between groups. However, the trajectory of PANSI-PSI scores did not differ between the ketamine and midazolam groups (*P* = .077) (Figure 4). Finally, between (ketamine vs midazolam)-group adverse effects, including derealization (n = 29 vs 7, *P* < .001), dizziness (n = 24 vs 5, *P* < .001), and crying (n = 6 vs 0, *P* = .026), were noted only during infusion and totally remitted at 80 minutes post infusion (Table 1). Finally, but importantly, there were 3 serious events of attempted suicide (drug overdose) in our clinical trial: 1 patient (on day 10 post infusion) in the ketamine group and 2 patients (on day 8 and day 12 post infusion, respectively) in the midazolam group. They were hospitalized for crisis intervention.

## DISCUSSION

Our results suggest that the antidepressant effect of low-dose ketamine infusion persists for 14 days in patients with TRD and prominent suicidal ideation. However, based on clinician-rated and self-reported measures, the antisuicidal effect of the ketamine infusion may diminish after 5–7 days. Additionally, our study highlights how treatment timing influences the antidepressant and antisuicidal effects of low-dose ketamine; only patients with moderate or low treatment refractoriness, those whose current depressive episode had lasted <24 months, and those with ≤4 failed antidepressant treatments benefited from the low-dose ketamine infusion.

Previous studies have investigated the associations between treatment refractoriness and the response to conventional antidepressants, prefrontal theta-burst stimulation (TBS), and electroconvulsive therapy (Fekadu et al., 2009; Ruhe et al., 2012; Li et al., 2014; van Diermen et al., 2018). Fekadu et al. identified the number of prior failed antidepressant treatments, the duration of the current depressive episode, and the severity of depressive symptoms as predictive factors of treatment response to antidepressants (Fekadu et al., 2009). In a study on prefrontal TBS in patients with TRD, only patients with low or moderate treatment refractoriness benefited from 10 sessions of TBS treatment (Li et al., 2014). Furthermore, our previous clinical trial of ketamine vs normal saline infusion in 8 patients with TRD and severe treatment refractoriness revealed no antidepressant effect of ketamine (Chen, Lin et al., 2021a). In the present study, we enrolled 41 patients with severe treatment refractoriness and revealed no antidepressant or antisuicidal effects of low-dose ketamine in such patients, corroborating our previous findings (Chen, Lin et al., 2021a).

Treatment refractoriness lies on a clinical spectrum; refractoriness may include the failure of 3 antidepressants to that of all available antidepressants, depressive episodes lasting 6 months to more than 2 years, and response or resistance to neurostimulation (Fekadu et al., 2009). In the present study, only the patients with TRD whose current depressive episode had lasted <24 months and those with ≤4 failed antidepressant treatments benefited from the antidepressant and antisuicidal effects of low-dose ketamine. The antidepressant effect persisted for up to 2 weeks, but the antisuicidal effect lasted only 5 days, supporting the findings of Wilkinson et al. and Alnefeesi et al. (Wilkinson et al., 2018; Alnefeesi et al., 2022).

Based on our findings, we propose the following 2 clinical recommendations. First, clinicians should optimize their timing of low-dose ketamine treatment from patients with TRD on the basis of the disease and treatment course. Specifically, patients experiencing depressive episodes that have persisted ≥12 but <24 months or who have failed to respond to >2 but ≤4 antidepressants may benefit the most from ketamine infusion. Second, our previous study suggested that the symptoms of clinical depression can be ameliorated by biweekly ketamine infusion but that the inflammatory profiles of interleukin-2 and tumor necrosis factor-α may be improved more by weekly infusion (Chen,Wu et al., 2021b). However, for suicide prevention, we suggest biweekly ketamine infusion for patients with TRD and prominent suicidal ideation because the antisuicidal effect persists only 5 days.

Our application of the PANSI-PSI and PANSI-NSI may help to clarify the effect of low-dose ketamine on suicidal symptoms (Osman et al., 1998). The PANSI-PSI assesses protective factors (i.e., feeling life is worth living, feeling in control of one’s own life, and having confidence in future plans) related to suicidal symptoms, whereas the PANSI-NSI examines risk factors (i.e., considering suicide, feeling hopeless, feeling like a failure, feeling lonely or sad, wanting to end the pain) associated with suicidal symptoms (Osman et al., 1998). Our findings suggest that the antisuicidal effect of low-dose ketamine infusion is mainly driven by a reduction in negative ideation associated with suicide rather than an increase in positive ideation associated with life.

Finally, the inpatient setting was conducted in previous esketamine or ketamine clinical trials of patients with major depressive disorder and suicidal thoughts (Murrough et al., 2015; Grunebaum et al., 2018; Fu et al., 2020; Ionescu et al., 2021). In the clinical trial by Murrough et al., study inclusion was initially limited to inpatients (n = 10); the protocol was later changed to enable outpatients (n = 14) in order to improve research feasibility and generalizability (Murrough et al., 2015). However, we conducted our clinical trial in an outpatient setting with frequent in-person follow-up in the first week (days 2, 3, 5, and 7). Three serious events of attempted suicide were noted in our clinical trial. Our results may also echo the findings of Murrough et al. that there was no main effect of setting (outpatient vs inpatient) and no setting by treatment interaction on ketamine’s antisuicidal effect (Murrough et al., 2015).

Our study has several limitations. First, we examined ketamine as an add-on medication, meaning that the other medications used by the patients with TRD were not discontinued during the study period. The add-on study design is ethically appropriate for patients with TRD and prominent suicidal ideation and may provide more realistic data. Second, we administered only a single ketamine infusion to each patient. Further studies using repeated infusions of low-dose ketamine are warranted to examine the sustained antidepressant and antisuicidal effects of ketamine in patients with severe depression and suicidal ideation.

## CONCLUSIONS

In summary, our study results support the rapid antidepressant and antisuicidal effects of low-dose ketamine infusion on patients with TRD and prominent suicidal ideation. The antidepressant effect of ketamine persisted for up to 2 weeks, but the antisuicidal effect lasted only 5 days. In addition, timing of ketamine treatment is crucial; specifically, patients with moderate or low treatment refractoriness, current depressive episodes that have persisted <24 months, or ≤4 failed antidepressant treatments may receive the greatest benefits from low-dose ketamine infusion.

## Supplementary Material

pyad014_suppl_Supplementary_Figure

pyad014_suppl_Supplementary_Table

pyad014_suppl_Supplementary_Data

## Interest Statement

None of the authors in this study had any conflict of interest to declare.
