# Supplementary figures and images for: A Randomized, Double-Blind, Midazolam-Controlled Trial of Low-Dose Ketamine Infusion in Patients With Treatment-Resistant Depression and Prominent Suicidal Ideation
Source: Int J Neuropsychopharmacol. 2023 Mar 26;26(5):331–9. doi: 10.1093/ijnp/pyad014 (PMC10229851; doi:10.1093/ijnp/pyad014)

Supplementary figure 1. Study flowchart


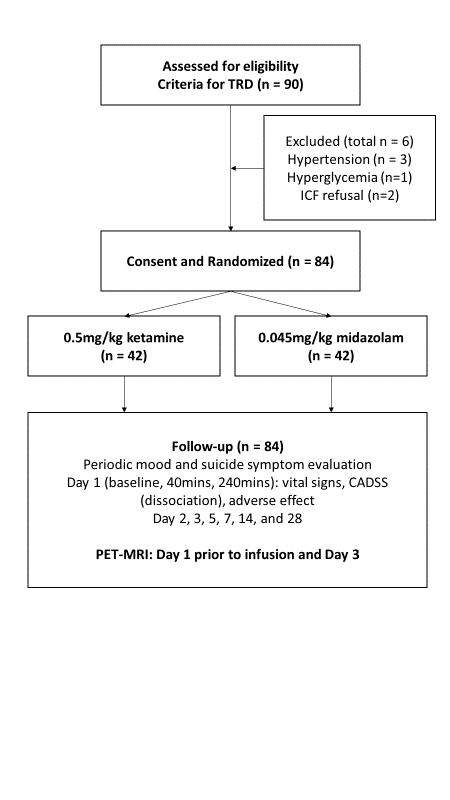

Supplement: pyad014_suppl_Supplementary_Figure [file pyad014_suppl_supplementary_figure.doc]
