# Supplementary material for: A Randomized, Double-Blind, Midazolam-Controlled Trial of Low-Dose Ketamine Infusion in Patients With Treatment-Resistant Depression and Prominent Suicidal Ideation
Source: Int J Neuropsychopharmacol. 2023 Mar 26;26(5):331–9. doi: 10.1093/ijnp/pyad014 (PMC10229851; doi:10.1093/ijnp/pyad014)
Supplement: pyad014_suppl_Supplementary_Table [file pyad014_suppl_supplementary_table.doc]

Supplementary table 1. Adverse effects during infusion

|  | Patients with TRD having prominent suicidal ideation | |  |
| --- | --- | --- | --- |
|  | Ketamine group (n=42) | Midazolam group (n=42) | p-value |
| Derealization (n, %) | 29 (69.0) | 7 (16.7) | < 0.001 |
| Dizziness (n, %) | 24 (57.1) | 5 (11.9) | < 0.001 |
| Nausea (n, %) | 2 (4.8) | 4 (9.5) | 0.676 |
| Crying (n, %) | 6 (14.3) | 0 (0.0) | 0.026 |
| Somnolence (n, %) | 1 (2.4) | 0 (0.0) | >0.999 |
